# Primary intracranial squamous cell carcinoma arising in epidermoid cysts: A case report and review of literature

**DOI:** 10.1097/MD.0000000000042094

**Published:** 2025-04-18

**Authors:** Tao Lin, Ling Ding

**Affiliations:** a Department of Neurosurgery, Shandong Second Provincial General Hospital, Jinan, Shandong, China; b Department of Otorhinolaryngology-Head and Neck Surgery, Shandong Provincial Hospital affiliated to Shandong First Medical University, Jinan, Shandong, China.

**Keywords:** intracranial epidermoid cyst, malignant transformation, primary intracranial squamous cell carcinoma

## Abstract

**Rationale:**

A natural malignant transformation of intracranial epidermoid cysts (IECs) into squamous cell carcinomas (SCCs) is very rare, and the mechanism remains unclear. Due to its rarity, there is currently no consensus on the management of primary intracranial SCC (PISCC) at present.

**Patient concerns:**

A 55-year-old woman was with an IEC in the right cerebellopontine angle region in our report. Her neurological symptom deteriorated rapidly in the 1 month before the admission.

**Diagnoses:**

The preoperative diagnosis was IEC. Postoperative pathological examination confirmed the presence of SCC arising within the background of an epidermoid cyst.

**Interventions:**

Following a comprehensive preoperative assessment, including physical examination, laboratory tests, and imaging studies, the patient underwent subtotal resection of the lesion. Postoperatively, a pathological examination was performed on the lesion tissue. A follow-up assessment of the patient was conducted 1 year later. Based on this case, we reviewed pertinent literature and preliminarily investigated the optimal management strategy for PISCC.

**Outcomes:**

After treatment, the patient’s dizziness resolved, and she was discharged in good condition. One year later, subsequent magnetic resonance imaging scans demonstrated no evidence of lesion recurrence.

**Lessons:**

In practice, it is prone to make a misdiagnosis or fail to reach a diagnosis for PISCCs due to the absence of specific clinical characteristics. Our clinicians should be more conscious of the necessity for timely diagnosis through targeted clinical strategies, which would result in more targeted treatment and a better prognosis.

## 1. Introduction

Intracranial epidermoid cysts (IECs) are benign tumors that account for about 0.2% to 1.8% of all brain tumors.^[1,2]^ IECs are widely perceived as congenital lesions originating from misplaced heterotopic ectodermal cells in the neural tube at 3 to 5 weeks of embryonic development,^[3,4]^ and there is an extremely rare possibility for these cysts to transform into squamous cell carcinoma (SCC).^[5]^ The first case of such malignant transformation was reported in 1912 by Ernst.^[3]^ It was not until 1981 when Garcia et al systematically established the criteria for identifying the malignant transformation of IECs for the first time (Table 1).^[6]^

**Table 1:** Criteria for the diagnosis of malignant transformation of IECs defined by Garcia et al and Hamlat et al.

Garcia criteria	
1	Tumor restricted to the intracranial, intradural compartment
2	Without extension beyond the dura, cranial orifices
3	Without connection with the middle ear, air sinuses, or sella turcica
4	No evidence of a nasopharyngeal tumor
Hamlat additional criteria	
5	Presence of a benign squamous cell epithelium within the malignant tumor
6	Exclusion of metastatic carcinoma

IEC = intracranial epidermoid cyst.

Twenty-four years later, in 2005, Hamlat and his colleagues further improved the diagnostic criteria (Table 1) and distinguished 5 types of the intracranial SCCs (Table 2).^[5,7]^ Since then, there had been unified criteria for the diagnosis and classification of PISCCs. Here, we presented a patient with initial malignant transformation of an epidermoid cyst into a SCC, discussed the treatment strategies and our clinical considerations. This would provide valuable ideas for clinical practitioners when faced with similar cases in the future. The patient in our case met all the criteria proposed by previous authors, supporting the diagnosis of primary intracranial squamous cell carcinoma (PISCC), while excluding the possibility of secondary SCC.

**Table 2:** Five types of the intracranial SCCs defined by Hamlat et al.

1	Initial malignant transformation of a benign cyst
2	Malignant transformation from a remnant cyst
3	Malignant transformation of a dermoid and epithelial cyst
4	Malignant transformation with leptomeningeal carcinomatosis
5	Other malignancies arising from benign cysts

SCC = squamous cell carcinoma.

## 2. Case presentation

**Figure 1.**
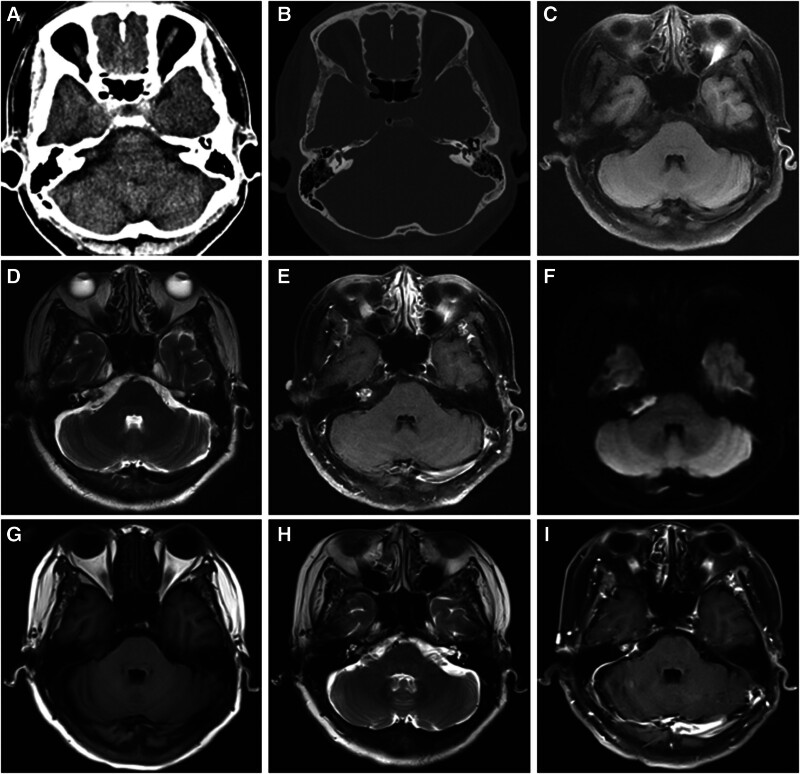
Preoperative images (A–F) and postoperative images at 1 year (G–I) were presented. Head axial CT images showed a mixed density expansive lesion of the right CPA region (A) and there was no significant bone damage to the internal auditory meatus on the affected side (B). T1- and T2-weighted imaging demonstrated a cyst and solid lesion with heterogeneous signals located in the right CPA region (C, D). T1-weighted gadolinium-enhanced MRI exhibited obvious mass-like enhancement in the solid part and hypointensity in the cystic part (E), while DWI showed an opposite signal (F). An anomalous signal was detected in the right internal auditory canal, presenting with slightly hypointense signal on T1 (G) and a mixed slightly hyperintense signal on T2 (H), as well as uneven enhancement on enhanced scan (I). CPA = cerebellopontine angle, CT = computed tomography, DWI = diffusion-weighted imaging, MRI = magnetic resonance imaging.

The patient, a 55-year-old woman, presented with a 1-month history of dizziness. She had a previous history of suspected benign positional vertigo and had sought treatment from the otolaryngology department. Following several tests, she received a diagnosis of an intracranial epidermoid cyst (IEC). Her eye movements and facial sensations were normal, and there were no signs of hyperalgesia resembling trigeminal neuralgia. Furthermore, no indications of facial nerve dysfunction, such as shallow frontal wrinkles, a crooked mouth, or incomplete eyelid closure, were observed. Audiological assessments revealed normal hearing in both ears. Further neurological examinations yielded normal results as well. Laboratory tests indicated a positive result for syphilis specific antibody Treponema pallidum particle agglutination assay, with no other abnormalities detected. She underwent a computed tomography scan revealing a mixed density extra-axial expansive lesion of the right cerebellopontine angle (CPA) region (Fig. 1A). In contrast to the normal side, there was no significant bone damage to the internal auditory meatus on the affected side (Fig. 1B). T1- and T2-weighted magnetic resonance imaging (MRI) (Fig. 1C, D) demonstrated a cyst and solid lesion with heterogeneous signals located in the right CPA region. T1-weighted gadolinium-enhanced MRI exhibited obvious mass-like enhancement in the solid part and hypointensity in the cystic part (Fig. 1E, arrows), while diffusion-weighted imaging showed an opposite signal (Fig. 1F).

**Figure 2.**
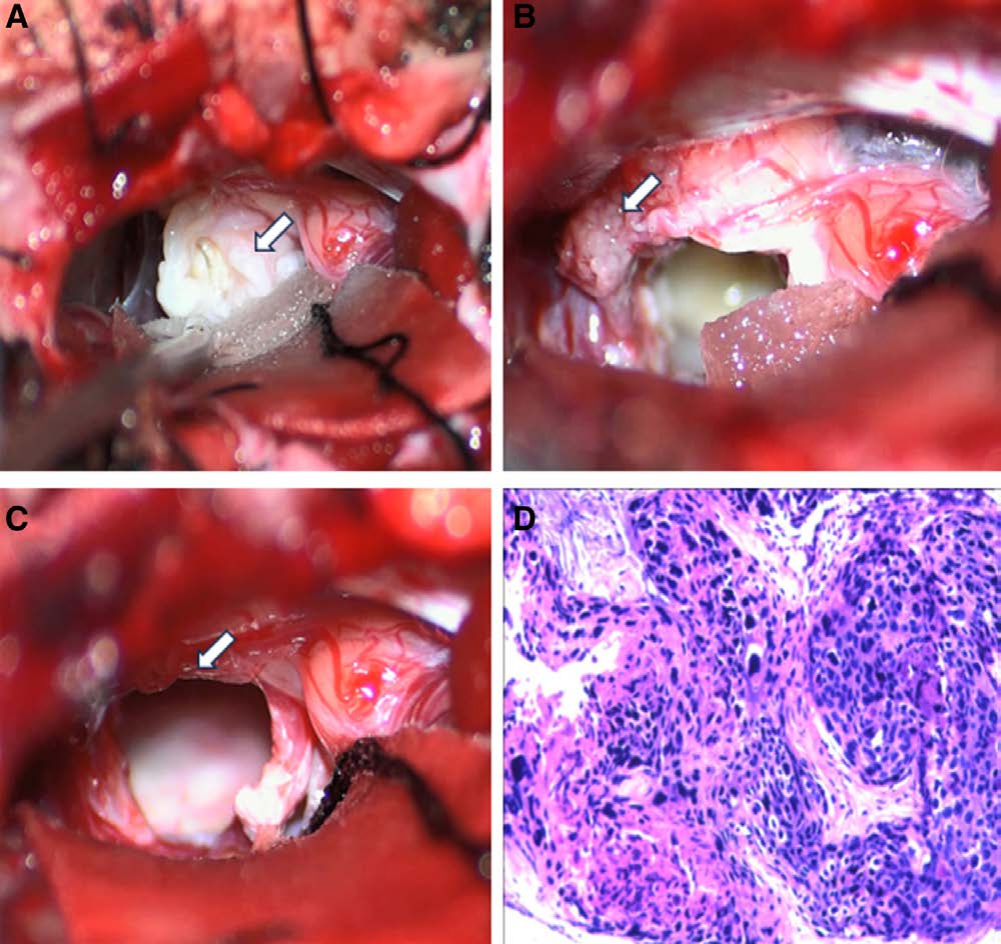
Intraoperative (A–C) and postoperative pathological (D) images were provided. Intraoperative photographs exhibited the debris inside of the lesion was a pearl-like luster under the microscope light (A), the solid portion located near the opening of the inner auditory canal (B), and was completely removed towards the conclusion of the surgical procedure (C). Postoperative pathology revealed a moderately differentiated squamous cell carcinoma with necrosis and keratinization (D). The tumor cells were positive for p63, P40, CK5/6, CK7, P53 immunostain, and Ki-67 index 35% (D).

A right suboccipital retrosigmoid approach was performed. Intraoperatively, the lesion was identified in the CPA region, outside of the brain tissue, and it had an intact capsule that enclosed adjacent nerves or vessels. Dissecting the cyst wall proved challenging as it was tightly adhered to the brainstem, basilar artery, auditory nerve, and facial nerve. After incising the wall, the debris inside exhibited a pearl-like luster under the microscope light (Fig. 2A, arrows). It lacked a blood supply, had a soft consistency, and could be easily aspirated. The solid portion of the tumor located near the opening of the inner auditory canal, appeared reddish, had a slightly tough texture (Fig. 2B, arrows) and was completely removed (Fig. 2C, arrows). This part had a rich blood supply and tight adhesion to the facial and auditory nerves. Towards the conclusion of the surgical procedure, the debris within the cyst was aspirated, while membranous tissue closely adjacent the brainstem, nerves, and blood vessels still remained partially (Fig. 2C). The tumor’s space-occupying effect was greatly alleviated.

The patient did not experience any evident postoperative headache or dizziness, and her body temperature and blood pressure remained within normal ranges. Apart from limited abduction of the right eye, she exhibited no signs of hearing impairment, facial paralysis, or additional neurologic dysfunction. Postoperative pathological examination revealed a squamous cell carcinoma on a background of IEC (Fig. 2D). The surgical incision healed satisfactorily, and the patient was discharged after removing stitches. Adjunctive radiotherapy was recommended. However, the patient declined due to personal reasons. One-year post-surgery, MRI revealed there was an anomalous signal in the right internal auditory canal, presenting with slightly hypointense signal on T1 and a mixed slightly hyperintense signal on T2, as well as uneven enhancement on enhanced scan (Figs. 1G–I).

## 3. Discussion

PISCC arising in ES is very rare and mostly found secondary to malignant transformation from a remnant cyst after surgery.^[3,5,8]^ A natural malignant transformation of IECs into SCCs is even rare, and the mechanism remains unclear.^[8–10]^ There is a view that chronic inflammation caused by repeated cyst rupture or subtotal resection of the cyst wall may predispose to the development of squamous cell carcinoma.^[4,5]^ In another argument, there is probably some truth that carcinoma in situ has existed in the squamous epithelium of epidermoid cyst, which may occur malignant transformation after prolonged evolution.^[5,10–12]^

The clinical signs and symptoms of patients with PISCC are not specific. Based on available literature, it is crucial to highly suspect malignant transformation in patients with IECs if they experience rapidly worsening symptoms or signs.^[4,13,14]^ In this particular case, the patient had been asymptomatic for a prolonged period. However, after experiencing dizziness for only 1 month, the diagnosis of a PISCC was made. To our knowledge, this was the shortest interval between the onset of illness and diagnosis. The importance of early diagnosis cannot be underestimated in terms of treatment planning and patient prognosis. Nevertheless, it is easy to make a misdiagnosis or miss a diagnosis in clinical practice due to the lack of specific imaging characteristics for PISCCs. As shown in this case, on MRI, the lesion was cystic and solid in the ostium of the internal auditory meatus, which was difficult to distinguish from acoustic neuroma or IEC. Preoperative temporal bone computed tomography, which enabled a clear visualization of bone near the inner auditory meatus, was inconclusive as to whether it was helpful in the differential diagnosis of each. The surgeons’ capacity to accurately differentiate between benign and malignant lesions during surgical procedures is also of great importance. During the procedure, we found that the solid part was significantly abnormal, so we removed as much tissue as possible from this part. The surgical results were satisfactory. The MRI conducted 1-year post-surgery revealed no significant recurrence of SCC (Figs. 1G–I).

Due to its rarity, there is no consensus on the management of PISCC at present. Here, based on relevant literature and combined with our clinical practice, we summarized the widely accepted diagnostic and therapeutic clinical strategies for PISCC, hoping to assist physicians who may encounter this disease. Patients with IEC who are asymptomatic in the early stage but deteriorate rapidly once symptoms appear should be deeply suspected as SCC patients.^[4,14]^ Enhanced MRI is indispensable after IEC diagnosis.^[4,12,15]^ The malignant transformation of IECs exhibit enhancement on contrast T1-weighted MRI. Surgeons should be particularly wary of abnormally enhanced tissue masses in cysts.^[16]^ The clinical presentation of the patient is still essential to judge the tissue origin of the tumor. For example, patients with acoustic neuroma generally exhibit tinnitus and hearing loss, while those with meningioma commonly experience prolonged headaches and dizziness. During the operation, it is recommended to remove the apparently abnormal tissue mass as thoroughly as possible^[15]^ and to perform a pathological examination to further confirm the nature of the tissue.^[16]^ Postoperative concurrent chemotherapy and radiotherapy should be performed in time when SSC is diagnosed, because the integration of multiple therapeutic modalities provides a superior prospect of survival compared to the efficacy of each modality independently.^[4,8,13,14]^ Close postoperative follow-up is still recommended.^[3,12,13]^ However, more evidence is needed to ascertain the optimal approach for managing this disease.

## 4. Conclusions

In this paper, we conducted a retrospective analysis of the comprehensive treatment process for a PISCC patient and summarized strategies for enhancing PISCC management based on pertinent existing literature. Our research would support surgeons in making informed decisions during surgical procedures.

## Acknowledgments

The authors appreciate the patient and her family for allowing them to publish this report.

## Author contributions

**Conceptualization:** Tao Lin.

**Data curation:** Tao Lin, Ling Ding.

**Investigation:** Tao Lin, Ling Ding.

**Methodology:** Tao Lin, Ling Ding.

**Project administration:** Tao Lin, Ling Ding

**Resources:** Tao Lin.

**Software:** Tao Lin, Ling Ding.

**Supervision:** Ling Ding.

**Validation:** Ling Ding

**Writing – original draft:** Tao Lin.

**Writing – review & editing:** Ling Ding.
